# Schmidt´s syndrome found by tan: a case report

**DOI:** 10.11604/pamj.2024.48.53.35130

**Published:** 2024-06-10

**Authors:** Gabriela Venade, Catarina Almeida, Nídia Oliveira, Luis Costa Matos

**Affiliations:** 1 Internal Medicine Department, Centro Hospitalar Tondela Viseu, Viseu, Portugal

**Keywords:** Schmidt´s syndrome, autoimmune polyglandular syndrome, Addison’s disease, case report

## Abstract

Addison´s disease can form part of type 2 autoimmune polyglandular syndrome. The article reports the case of a 41-year-old female patient with hypothyroidism and vitiligo, who came to the emergency department complaining of asthenia that had worsened in recent months, as well as anorexia, nausea, and weight loss (6 kg in a year). Cutaneous hyperpigmentation was the main finding on physical examination, together with vitiligo lesions on the face, hands, and armpits. Further study revealed a low serum cortisol level, normal urine-free cortisol, and an elevated adrenocorticotropic hormone (ACTH). Antiperoxidase antibodies and 17-alpha-hidroxylase antibodies were both positive. Treatment was started with prednisolone and fludrocortisone, and a good clinical response was obtained. This case report aims to draw attention to the high level of clinical suspicion required to diagnose Addison´s disease and the need to screen actively for other potentially associated autoimmune diseases that may be associated.

## Introduction

Schmidt syndrome, also known as type-2 autoimmune polyglandular syndrome (APS-2), is one of a rare group of polyendocrine conditions that include multiple glandular deficiencies associated with other autoimmune diseases [1]. Schmidt´s syndrome is the combination of Addison´s disease, autoimmune thyroid disease, and/or type-1 diabetes mellitus [2]. Other autoimmune disorders, such as vitiligo, can also be associated with it [3].

Schmidt´s syndrome is rare, with around 1.4 - 4.5 cases per 100,000 inhabitants [4]. It is more prevalent among middle-aged women [4].

This report describes a case of APS-2 in a woman who had been diagnosed previously with hypothyroidism of unknown etiology combined with vitiligo. We diagnosed Addison's disease and the autoimmune origin of the hypothyroidism, stressing the need for clinical suspicion and the inclusion of potential multiple autoimmune disorders in a single diagnosis.

## Patient and observation

**Patient information:** a 41-year-old female patient with a history of hypothyroidism of unknown etiology combined with vitiligo presented at the emergency department complaining of asthenia that had worsened in recent months, together with anorexia, nausea, and weight loss of 6 kg in a year. Levothyroxine 112 µg and oral iron supplementation were the only medications she was taking.

**Figure 1:**
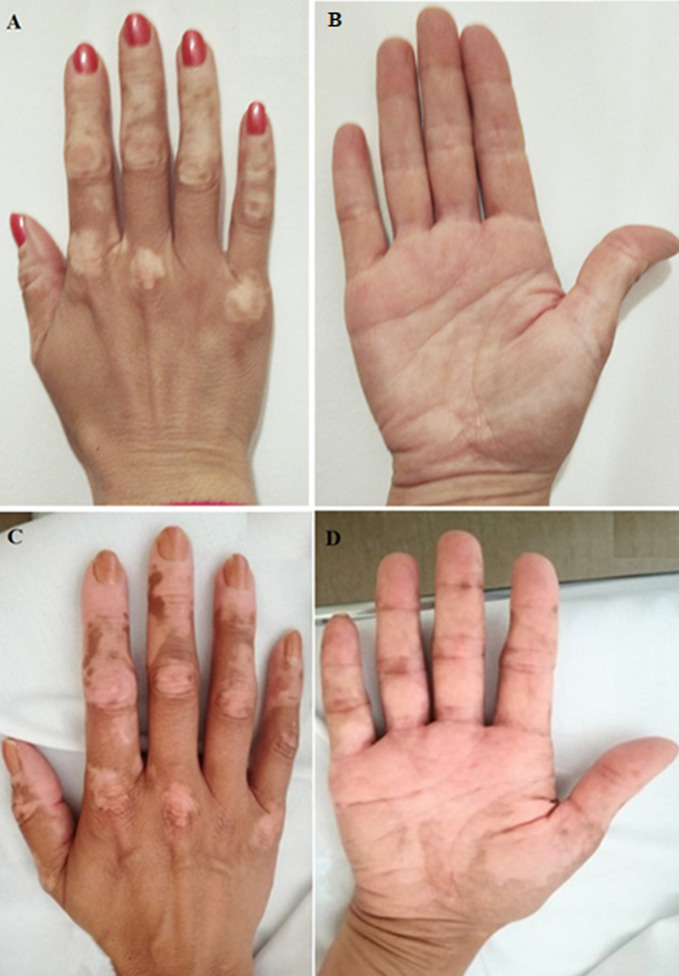
normal skin hyperpigmentation of the hands of patient contrasting with vitiligo of the right hand before the diagnosis of Addison´s disease: A) dorsum; B) palm; C,D) tanned skin hyperpigmentation of the hands in the dorsum and palm during the diagnosis of Addison´s disease

**Clinical findings:** on physical examination, apart from vitiligo lesions on the face, hands, and armpits, the patient presented cutaneous hyperpigmentation (tanning) (Figure 1). She also had a hypotensive profile (106/77 mmHg). No other positive discoveries were detected in the physical exam.

**Timeline of the current episode:** in April 2019, the patient was evaluated in the emergency department and blood samples were collected and the patient was hospitalized for further study and diagnostic workup. She was discharged ten days after admission and re-evaluated in May 2019.

**Diagnostic assessment:** discrete hyponatremia (Na 133 mEq/L), hyperkalemia (K 4.8 mEq/L) with no changes in leucocytes or hemoglobin (Table 1). Serum cortisol was 3.3 µg/dL (4.3 - 22.4 µg/dL), urine-free cortisol 52 µg/24h (28-214 µg/24h), ACTH > 2000.0 pg/mL (4.7-48.8 pg/mL) (Table 2). QuantiFERON TB test was negative and hemorrhage, infiltrates, or masses in the adrenal ultrasound were also excluded. Antiperoxidase antibodies were positive. The 21-hydroxylase antibody test was negative but the 17-alpha-hidroxylase antibody test was positive. HbA1c was 5.1%, anti-transglutaminase and anti-endomysial antibodies were all negative.

**Table 1:** general analytical results of blood samples collected at the emergency department based on patient complaints

	Value	Reference value
Leucocytes	4.70 x 109/L	4.50 - 11.50 x109/L
Hemoglobin	12.8 g/dL	12.0 - 15.0 x109/L
Platelets	236.0 x 109/L	150.0 - 450.0 x109/L
Sodium	133 mEq/L	136 - 145 mEq/L
Potassium	5.0 mEq/L	3.4 - 4.4 mE1/L
Calcium	4.8	4.2 - 5.1
Albumin	3.8	3.5 - 5.0
Urea	65	14 - 42
Creatinine	1.0 mg/dL	0.5 - 1.2 mg/dL
LDH	345 UI/L	200 - 480 UI/L

LDH: lactate dehydrogenase

**Table 2:** specific analytical results of blood and urine collected during the diagnostic workup when the patient was admitted to the internal medicine department, allowing Addison disease diagnosis and also autoimmune polyglandular syndrome type 2

	Value	Reference value
Anti-thyroid peroxidase antibodies	1196.6 UI/mL	0.0 - 60.0
Anti-thyroglobulin antibodies	<15.0 UI/mL	0.0 - 60.0
A1c hemoglobin	5.1%	4.0 - 6.5
Anti-transglutaminase IgA antibodies	0.50 AU/mL	0.00 - 10.00
Anti-transglutaminase IgG antibodies	0.10 GU/mL	0.00 - 10.00
Morning Serum cortisol	3.3µ g/dL	4.3 - 22.4
Urinary cortisol	52 µg/24 h	28 - 214
ACTH	>2000.0 pg/mL	4.7 - 48.8
Anti-endomysial IgA antibodies	Negative	
Anti-endomysial IgG antibodies	Negative	
Quantiferon TB	Negative	
21-hydroxylase antibody	Negative	
17-alpha-hidroxylase antibody	Positive	

ACTH: adrenocorticotropic hormone

**Diagnosis:** the diagnosis of an autoimmune polyglandular type 2 syndrome with autoimmune hypothyroidism and Addison’s disease was established.

**Therapeutic interventions:** daily treatment was started with 7.5 mg of prednisolone and 0.1 mg of fludrocortisone.

**Follow-up and outcome of interventions:** the patient presented a good clinical response after starting treatment, regaining her previous health status as self-reported.

**Patient perspective:** “since I´ve been taking this new medication, I´ve started to feel better, with improvements in asthenia, and anorexia; and I´ve regained my normal skin colour”.

**Informed consent:** the patient has provided informed consent to the publication of this case report. She has also collaborated, generously and cooperatively, in the photographic documentation of her lesions, having been made aware of the intention to publish.

## Discussion

There are three autoimmune polyglandular syndromes (APS), of which type 2 (APS2) is the least rare, with around 1.4 - 4.5 cases /100000 inhabitants [4].

The presence of Addison´s disease combined with autoimmune thyroid disease and/or type 1 diabetes mellitus defines this syndrome [4]. Other minor autoimmune disorders can also be concomitant, but are not included among the diagnostic criteria. An example is vitiligo [3] which our patient also presented. The disease is more prevalent among middle-aged women, such as our patient. Addison´s disease with Hashimoto´s thyroiditis is clinically the most frequent combination and accounts for the majority of cases. Addison´s disease is usually the first to be diagnosed and thyroiditis later [4,5]. Although our patient presents the most frequent form of APS2 combination, the diseases appeared in the reverse order.

Early diagnosis of Addison´s disease (AD) significantly reduces its morbidity and mortality. AD is the main cause of adrenal insufficiency and forms part of an APS in 60% of cases [6,7]. The onset of the disease can be insidious and the clinical presentation nonspecific, which complicates and delays the diagnosis. Hyperpigmentation and salt-craving are highly specific indicators of primary adrenal insufficiency [6]. Our patient had a nonspecific clinical presentation and it was hyperpigmentation of her skin that led us to suspect Addison´s disease and, then, APS2.

In APS´s cases, patients with one autoimmune disease are more likely than the population at large to develop a new autoimmune disease. Circulating antibodies associated with a particular type of disease may exist months to years before the disease develops [4]; hence, the importance of screening for other disorders when an autoimmune pathology is diagnosed.

This case highlights the need for a high degree of clinical suspicion when diagnosing Addison´s disease. The photographic documentation of the skin hyperpigmentation in a part of the body that also displays vitiligo facilitates recognition of this nonspecific clinical presentation of AD.

Given its rarity, it is essential to screen for other potential autoimmune diseases with the aim of ruling out APS2, as with our patient who presented vitiligo and hypothyroidism of unknown aetiology.

## Conclusion

Autoimmune polyglandular syndromes type 2 (APS2) is a rare condition that should be screened for in patients with one or more autoimmune diseases. The aim of this case report is to illustrate the importance of a high level of clinical suspicion when diagnosing Addison´s disease and the need to screen for other associated autoimmune diseases in order to rule out APS2.
